# The GPR88 Agonist RTI‐122 Reduces Alcohol‐Related Motivation and Consumption

**DOI:** 10.1111/adb.70058

**Published:** 2025-06-19

**Authors:** Dennis F. Lovelock, Wen Liu, Sami Ben Hamida, Victoria L. Cordero, Kalynn J. Van Voorhies, Marion Martin, Isabella Guimaraes Olmo, Emmanuel Darcq, Md Toufiqur Rahman, Mickael Naassila, Brigitte L. Kieffer, Chunyang Jin, Joyce Besheer

**Affiliations:** ^1^ Bowles Center for Alcohol Studies University of North Carolina at Chapel Hill Chapel Hill North Carolina USA; ^2^ INSERM UMR 1247 University of Picardie Jules Verne Amiens France; ^3^ INSERM UMR‐S 1329, Strasbourg Translational Neuroscience and Psychiatry University of Strasbourg Strasbourg France; ^4^ Center for Drug Discovery Research Triangle Institute Research Triangle Park North Carolina USA; ^5^ Department of Psychiatry University of North Carolina at Chapel Hill Chapel Hill North Carolina USA

## Abstract

GPR88, an orphan G protein‐coupled receptor primarily expressed in the striatum, has emerged as a potential target for treating alcohol use disorder (AUD) due to its role in modulating reward and motivational pathways. In this study, we investigated the effects of the GPR88 agonist RTI‐122 on alcohol intake and motivation to self‐administer alcohol under different conditions. In mice, RTI‐122 reduced alcohol consumption in a two‐bottle choice paradigm, which was prevented by *Gpr88* knockout, confirming a GPR88‐specific effect on the attenuation of alcohol drinking. In rats, RTI‐122 dose‐dependently reduced operant alcohol self‐administration and decreased motivation to self‐administer alcohol in progressive ratio tasks, regardless of whether the alcohol was adulterated with quinine or not. Additionally, a high dose of RTI‐122 reduced yohimbine‐induced reinstatement. Importantly, RTI‐122 did not affect water intake in mice or sucrose self‐administration in rats, indicating receptor‐ and reward‐specific modulation of alcohol intake. These findings suggest that RTI‐122, through GPR88 agonism, effectively reduces alcohol consumption and motivation across various contexts, positioning it as a promising lead for the development of new AUD treatments.

## Graphical abstract

In mice, RTI‐122, a GPR88 agonist, reduced alcohol consumption in a two‐bottle choice paradigm, which was prevented by Gpr88 knockout. In rats, RTI‐122 decreased alcohol self‐administration, reduced breakpoints in progressive ratio tasks with and without quinine adulteration and attenuated yohimbine‐induced reinstatement. These findings suggest RTI‐122 reduces alcohol drinking and motivation through GPR88 activation, positioning it as a promising lead for the development of new alcohol use disorder treatments.

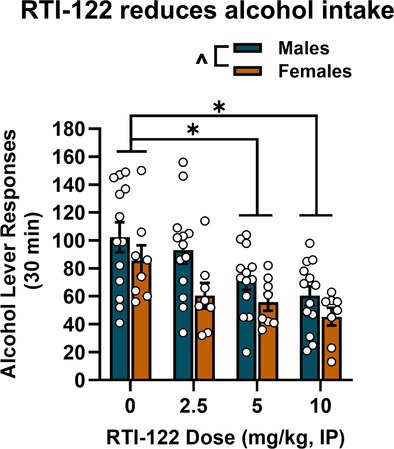

## 1 Introduction

Alcohol use disorder (AUD) is a significant public health issue, affecting approximately 29.5 million people in the United States as of 2022 [1]. Despite the availability of pharmacotherapeutic treatments for AUD, these medications have limitations including moderate efficacy with high variability between patients, side effects and adherence issues, with only one in six patients receiving treatment worldwide [2]. Despite this, it has been two decades since any drug has received FDA approval for the treatment of AUD, with Acamprosate having been approved in 2004. Thus, there is a great need for additional treatment options to address the varied needs and biological mechanisms of individuals suffering from AUD.

The G protein‐coupled receptor 88 (GPR88), a brain‐specific orphan receptor with high expression in the striatum [3, 4], has recently received growing attention as a druggable target for a range of psychiatric disorders [5]. *Gpr88* knockout (KO) studies in mice find deficits in learning, memory and reward processing [6, 7] as well as increased alcohol drinking and seeking [8]. Therefore, activation of this receptor may serve as a novel mechanism of treatment of substance use disorders and AUD. Towards this end, we have developed and characterized the first potent, selective and brain‐penetrant GPR88 agonist RTI‐13951‐33, which reduced binge‐like alcohol consumption and self‐administration in C57BL/6 mice [9, 10]. Notably, specificity was confirmed as it was ineffective in *Gpr88* KO mice. Subsequently, we improved the potency and pharmacokinetic properties by medicinal chemistry, identifying RTI‐122 as having greater metabolic stability and brain penetrance compared to RTI‐13951‐33 [11]. The in vivo efficacy of RTI‐122 was demonstrated through a drinking‐in‐the‐dark procedure in mice where it reduced alcohol consumption at a 10 mg/kg dose [11].

The present studies set out to provide a comprehensive characterization of RTI‐122 across multiple alcohol‐drinking models. First, we assessed the effects of RTI‐122 on general locomotor activity in male mice. Second, we conducted 20% alcohol two‐bottle choice intake in wild‐type (WT) and *Gpr88* KO mice, which confirmed a GPR88‐specific effect of RTI‐122. Third, we assessed alcohol‐reinforced drinking and reinforcer specificity using operant alcohol and sucrose self‐administration procedures in male and female alcohol‐preferring (P) rats. Next, we examined the efficacy of RTI‐122 to reduce motivation to self‐administer alcohol using a progressive ratio schedule and quinine adulteration to assess aversion‐resistant drinking. Lastly, to determine whether RTI‐122 may have efficacy in reducing relapse‐like behaviours, we assessed yohimbine‐induced reinstatement using a two‐phase procedure to measure seeking behaviour and reinitiation of drinking. This investigation demonstrates the efficacy of RTI‐122 in reducing alcohol consumption and motivation to drink across multiple procedures and in both sexes, supporting the therapeutic potential of GPR88 agonism as a target for AUD.

## 2 Materials and Methods

### 2.1 Subjects

#### 2.1.1 Mice

Male (WT or C57BL/6J) mice were acquired from Charles River, France for the locomotor activity experiment. For alcohol intake, male and female *Gpr88* ^−/−^ knockout mice were generated as previously described [9, 12] via germ‐line deletion of *Gpr88* exon 2 under a mixed background (13.96% C57B1/6; 60.94% C57B1/6J; 0.05% FVB/N; 25% 129/SvPas; 0.05% SJL/J). For experiments, mice were aged 3–5 months (25–35 g) and group‐housed (3–5/cage) under a 12‐h light/dark cycle (7:00 a.m./7:00 p.m.) or reverse 12‐h cycle (10:00 a.m.–10:00 p.m.) for two‐bottle choice experiments. All procedures were approved by the Regional Committee of Ethics in Animal Experimentation of Strasbourg (CREMEAS) and the Comité Régional d'Ethique en Matière d'Expérimentation Animale de Picardie (CREMEAP).

#### 2.1.2 Rats

Male and female alcohol‐preferring (P) inbred rats were bred on‐site at the University of North Carolina (UNC) at Chapel Hill and double‐housed under a 12‐h light/dark cycle (7:00 AM/7:00 PM), and separate groups were used for the self‐administration, progressive ratio and reinstatement experiments. Procedures followed the NIH Guide for the Care and Use of Laboratory Animals and were approved by the UNC Institutional Animal Care and Use Committee.

### 2.2 Drugs

RTI‐122, synthesized at Research Triangle Institute, was dissolved in saline (0.9%) and administered intraperitoneally (IP) (10 mL/kg for mice, 1 mL/kg for rats). Quinine (Sigma, Q1125) was diluted in 15% ethanol, and yohimbine (Sigma, Y3125) was prepared in 0.9% saline. For the self‐administration sessions, alcohol (95% (v/v), Pharmaco‐AAPER, Shelbyville, KY, cat. no. 04355226EA), and sucrose were diluted with tap water.

### 2.3 Experiment 1: Locomotor Activity (Mice)

Mice were placed in VersaMax chambers (Omnitech, USA) for a 15‐min habituation period, followed by a 60‐min postinjection session after RTI‐122 (2.5, 5, 10, 20 mg/kg, IP) or saline. Each mouse received one dose and one test. Distance travelled (cm) was recorded in 5‐min bins.

### 2.4 Experiment 2: Intermittent Access Two‐Bottle Choice (IA20%2bc, Mice)

Mice underwent 16 weeks of intermittent access to 20% alcohol as previously described [8, 9]. Mice had 24‐h concurrent access to 20% ethanol and water bottles on Monday, Wednesday and Friday. Intake was measured at 4 and 24 h postsession. RTI‐122 (0, 10, 20 mg/kg, IP) was injected 60 min before the drinking session. Male mice received both 10 and 20 mg/kg doses using a within‐subject design. Female mice were initially tested at 10 mg/kg in a within‐subject design, but after half the cohort had received either 10 mg/kg or vehicle with no observable effects, the remaining animals were not tested at that dose, resulting in a between‐subject comparison. All females were subsequently tested at 20 mg/kg using a within‐subject design.

### 2.5 Experiment 3: Alcohol and Sucrose Self‐Administration (Rats)

Self‐administration was conducted in 30 min sessions in individual operant chambers (Med Associates, Georgia, VT) within sound‐attenuating cabinets with an exhaust fan to provide ventilation and mask external noise [13, 14, 15]. Briefly, chambers were equipped with an active and inactive lever on opposite sides of the chamber (left and right). When the fixed ratio 2 (FR2) response requirement was met on the active lever (left), a cue light and stimulus tone were presented alongside alcohol or sucrose reinforcer delivery (0.1 mL) via a syringe pump. All responses were recorded, but responses on the inactive lever produced no programmed consequence. Four parallel infrared beams across the bar floor measured session locomotor activity; the number of beam breaks was divided by session length (30 min) as the locomotor rate (beam breaks/min). To induce stable and consistent alcohol intake, rats were trained to self‐administer 15% (v/v) alcohol (FR2 schedule, 30‐min sessions, 5 days/week) using a sucrose fading procedure [14, 15]. Once stable responding was established, RTI‐122 (0, 2.5, 5, 10 mg/kg, IP) was tested in a within‐subject Latin square design, with doses balanced across test days and sex (males: *n* = 13; females: *n* = 8). On these test sessions, RTI‐122 was injected 60 min prior to a self‐administration session. Test sessions occurred on Tuesdays and Thursdays, with standard self‐administration sessions on Monday, Wednesday and Friday. To assess reinforcer specificity, following alcohol testing, the same rats proceeded to 0.8% (w/v) sucrose self‐administration, with 8 days of training before RTI‐122 testing on sucrose self‐administration began. RTI‐122 doses and testing procedures were identical to the alcohol self‐administration testing.

### 2.6 Experiment 4: Progressive Ratio (Rats)

For progressive ratio (PR) testing, a separate cohort of rats was trained on alcohol self‐administration then began PR1 testing. Under the PR1 schedule, the response requirement for alcohol delivery increased by 1 each time a reinforcer was delivered (PR1: 1, 2, 3, 4, etc.). The breakpoint was defined as the highest ratio completed during the 30‐min session. RTI‐122 (0, 2.5, 5, 10 mg/kg, IP) was tested using a within‐subject Latin square design with at least one intervening self‐administration session between test sessions. After completion of testing, the same rats began evaluation of aversion‐resistant alcohol consumption using the same PR1 schedule with the exception that quinine‐adulterated alcohol (0.9 g/L) was the reinforcer on the tests. The effects of 0, 2.5 and 5 mg/kg RTI‐122 on motivation to self‐administer quinine‐adulterated alcohol were assessed in a within‐subject design (10 mg/kg was excluded due to suppression of unadulterated alcohol intake).

### 2.7 Experiment 5: Yohimbine‐Induced Reinstatement (Rats)

This study was conducted in a separate cohort of self‐administration trained rats. Following training, rats underwent 16 extinction sessions (30 min/session) where both active and inactive lever responses were recorded but produced no programmed consequence (i.e., no cues, no alcohol delivery). On reinstatement test days, rats received RTI‐122 (0, 10, or 20 mg/kg, IP), followed 30 min later by yohimbine (1.25 mg/kg, IP) administered in a within‐subject Latin square design. One hour post‐RTI‐122 injection, a 30‐min two‐phase reinstatement session was conducted, with the first 15 min as a seeking phase where FR2 responses triggered cue presentation without alcohol delivery, and the remaining 15 min was a reinitiation phase where FR2 responses led to both alcohol and cue delivery akin to standard self‐administration [16]. Extinction resumed between tests, requiring < 30 active lever responses across the three most recent sessions before retesting. See Figure S1.

### 2.8 Data Analysis

Locomotor activity was analysed using one‐way ANOVA with Dunnett's post hoc. For the two‐bottle choice experiments, data were analysed with two‐way ANOVA with or without repeated measures. Significant main effects and interactions of the ANOVAs were further investigated with the Bonferroni post hoc tests or method of contrast analysis. Statistical significance was set at *p* < 0.05. Analysis of the self‐administration, progressive ratio and reinstatement experiments used two‐ or three‐way ANOVA as appropriate. For extinction sessions, alcohol responses and locomotor rate were analysed using two‐way RM‐ANOVA (dose, sex). Bonferroni post hoc was applied where appropriate. Analyses used GraphPad Prism v10.

## 3 Results

### 3.1 Experiment 1: Locomotor Activity

**FIGURE 1:**
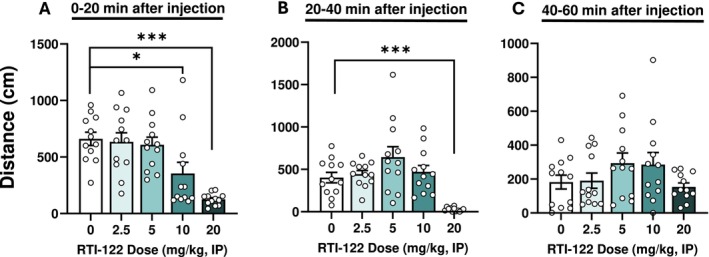
RTI‐122 dose‐dependently reduced spontaneous locomotor activity in WT male mice. (A) Total distance travelled during the first 0–20 min, (B) during 20‐ to 40‐min time periods and (C) during 40‐ to 60‐min time periods of the test. Four doses of RTI‐122 were tested (2.5, 5, 10 and 20 mg/kg). RTI‐122 at 20 mg/kg strongly reduced spontaneous locomotion during the first 0–20 min (A) and 20–40 min (B) periods. RTI‐122 at 10 mg/kg reduced spontaneous locomotion transiently during the first time period (A). The 2.5 and 5 mg/kg doses of RTI‐122 do not alter spontaneous locomotion. Importantly, after 40 min, RTI‐122 does not show any effect on spontaneous locomotion (C). Data are presented as average of total distance ± S.E.M. Vehicle vs. RTI‐122 *p < 0.05, **p < 0.01 and ***p < 0.001, *n* = 12/group.

RTI‐122 dose‐dependently reduced spontaneous locomotion in WT males during the first 20 min [F(4,55) = 10.97, *p* < 0.0001], with significant effects at 10 mg/kg (*p* < 0.001) and 20 mg/kg (*p* < 0.05; Figure 1A). During the 20–40 min period, only 20 mg/kg reduced locomotion [F(4,55) = 9.69, *p* < 0.0001] (*p* < 0.01 vs. vehicle; Figure 1B). No effects were detected during the 40–60 min period (Figure 1C). To prevent confounding general effects on alcohol‐drinking behaviour, all subsequent experiments in mice and rats were conducted 1‐h postadministration.

### 3.2 Experiment 2: Intermittent Access Two‐Bottle Choice

**FIGURE 2:**
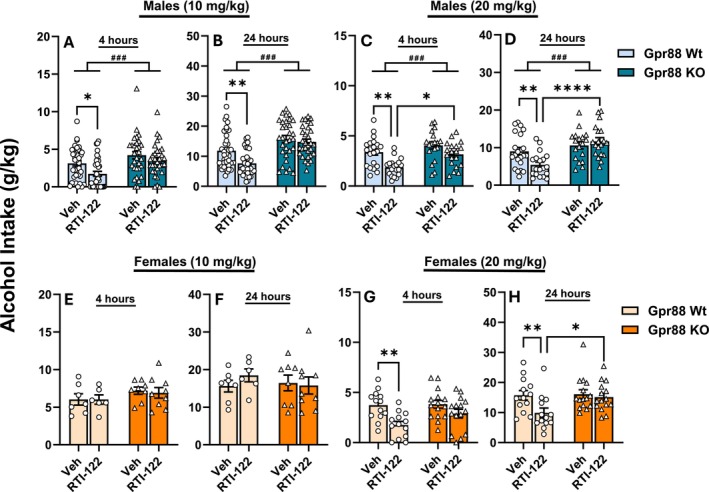
RTI‐122 reduced alcohol consumption in the two‐bottle choice test. *Gpr88* knockout (*Gpr88* KO) and control (*Gpr88* WT) mice underwent the IA20%‐2bc procedure for 6 weeks. On test days, male (A–D) and female (E–H) mice received either vehicle or RTI‐122 at doses of 10 mg/kg (males: A–B; females: E–F) or 20 mg/kg (males: C–D; females: G–H), administered 60 min prior to the start of the 24‐h session. Alcohol intake was measured at 4 and 24 h. Data are presented as mean ± S.E.M. A–B, *n* = 31–37; C–D, *n* = 18–21; E–F, *n* = 6–9 G–H, *n* = 13–15. Treatment effect: *p < 0.05; **p < 0.01; genotype effect: ##*p* < 0.01, ###*p* < 0.001.

To determine the in vivo specificity of RTI‐122, we examined its effect on intermittent access to 20% alcohol two‐bottle choice intake with *Gpr88* KO mice and their WT controls (Figure 2A–H) and overall found that *Gpr88* KO prevented RTI‐122‐induced reductions in alcohol intake that were seen in controls. No effects on water intake were detected (see Figure S2). In WT males, RTI‐122 (10 mg/kg) significantly reduced alcohol intake at both 4‐ and 24‐h time points [4 h (Figure 2A): F(1,66) = 6.96, *p* < 0.05; 24 h (Figure 2B): F(1,66) = 8.59, *p* < 0.01] with no reduction found in *Gpr88* KO males. *Gpr88* KO mice consumed more alcohol overall at both time points [4 h: F(1,66) = 14.61, *p* < 0.001; 24 h: F(1,66) = 31.23, *p* < 0.001]. At 20 mg/kg, the same patterns were observed as RTI‐122 also reduced alcohol intake at 4 h [Figure 2C: main effects of treatment F(1,73) = 16.86, *p* < 0.0001] and 24 h [Figure 2D: Significant treatment‐genotype interaction, F[1,73] = 6.4, *p* = 0.013], with a significant genotype effect [4 h: F(1,73) = 16.86, *p* < 0.01; 24 h: F(1,73) = 17.86, *p* < 0.0001]. Post hoc analyses confirmed that RTI‐122 significantly reduced alcohol intake in WT control males at all doses and time points (*p* < 0.05) and replicated previous findings that *Gpr88* KO males consume more alcohol than controls during 24‐h sessions (*p* < 0.05) [8, 9].

In WT control females, only the 20 mg/kg dose was effective, reducing alcohol intake at 4 h [Figure 2G: F(1,26) = 17.98, *p* < 0.0001] and 24 h [Figure 2H: F(1,26) = 7.5, *p* < 0.01], and RTI‐122 had no effect in *Gpr88* KO females. Post hoc analyses confirmed a significant reduction in alcohol intake (*p* < 0.01) in WT control females. Overall, these results suggest dose‐dependent sensitivity in females, as the 10 mg/kg dose did not significantly reduce alcohol intake. These findings indicate that the reductions in alcohol intake in control mice are likely mediated by GPR88 receptor activity, supporting the specificity of RTI‐122 as a GPR88 receptor agonist.

### 3.3 Experiment 3: Alcohol and Sucrose Self‐Administration

**FIGURE 3:**
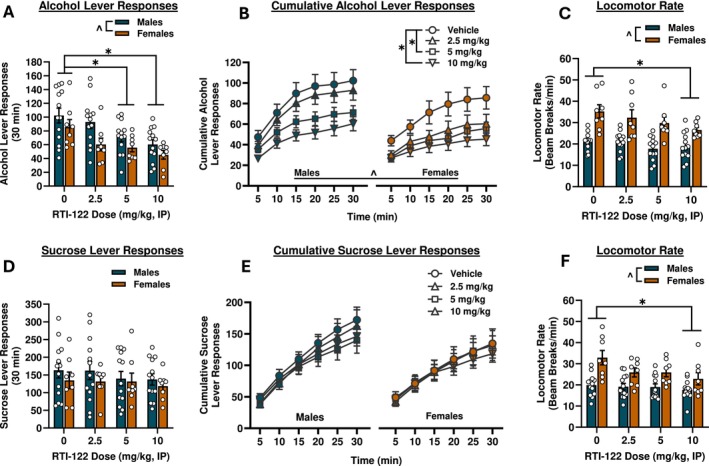
RTI‐122 reduced alcohol, but not sucrose, self‐administration. Doses of 5 and 10 mg/kg RTI‐122 reduced alcohol lever responses in 30‐min self‐administration sessions (A) and when data were split into 5‐min bins (B). The highest dose reduced locomotor rate during the session (C). RTI‐122 had no effects on sucrose self‐administration (D&E) and again the 10 mg/kg dose reduced locomotor rate (F). Males, *n* = 13; females, *n* = 8. *effect of treatment, ^effect of sex: *p* < 0.05.

As shown in Figure 3A, RTI‐122 reduced alcohol self‐administration in the P‐rats. Two‐way RM ANOVA found that RTI‐122 reduced alcohol lever responses [F(3,57) = 9.59, *p* < 0.0001] with post hoc analyses finding reductions at the 5 and 10 mg/kg doses (*p* < 0.05). Overall, females responded less than males [F(1,19) = 5.90, *p* < 0.05], though there was no interaction. RTI‐122 similarly reduced alcohol intake (see Table 1) [F(3,57) = 10.27, *p* < 0.0001], with reductions at the 5 and 10 mg/kg doses (*p* < 0.01). There was also an expected significant effect of sex as females consumed more alcohol relative to body weight (g/kg) than males [F(1,19) = 6.70, *p* < 0.05]. The three‐way RM ANOVA on cumulative alcohol lever responses across the session (Figure 3B) showed the main effects of sex [F(1,19) = 6.12, *p* < 0.05], dose [F(3,17) = 7.25, *p* < 0.05] and time [F(5,15) = 24.43, *p* < 0.001], with significant reductions at 5 and 10 mg/kg and males responding more than females overall (*p* < 0.01). Females exhibited a greater locomotor rate during the self‐administration testing sessions than males [F(1,19) = 15.72, *p* < 0.001], and there was an overall RTI‐122‐induced reduction in locomotor rate [F(3,57) = 5.35, *p* < 0.01] at the highest dose (10 mg/kg) only (*p* < 0.01). Responding on the inactive lever was very low, and no differences were found (Table 1).

**TABLE 1:** Intake and inactive lever responses from Experiment 3 and inactive lever response data from Experiments 4 and 5.

	Measure	Sex	RTI‐122 dose (mg/kg, IP)				
			0	2.5	5	10	20
Exp 3: Alcohol self‐administration	Intake (g/kg)	Males	1.29 ± 0.13	1.2 ± 0.13	**0.94 ± 0.09**	**0.78 ± 0.09**	
		Females	1.83 ± 0.20	1.35 ± 0.18	**1.24 ± 0.14**	**1.04 ± 0.15**	
	Inactive lever responses	Males	2.85 ± 0.72	2.15 ± 0.72	2.15 ± 0.65	2.31 ± 0.96	
		Females	9.38 ± 3.17	5.50 ± 1.94	5.75 ± 2.40	3.75 ± 1.73	
Exp 3: Sucrose self‐administration	Intake (g/kg)	Males	16.56 ± 2.15	17.41 ± 2.78	14.01 ± 1.97	13.87 ± 1.71	
		Females	22.11 ± 4.14	22.88 ± 2.81	23.03 ± 4.22	21.07 ± 2.35	
	Inactive lever responses	Males	1.85 ± 0.82	1.38 ± 0.46	0.92 ± 0.29	2.08 ± 0.85	
		Females	4.25 ± 2.01	4.25 ± 1.25	1.38 ± 0.38	2.38 ± 0.68	
Exp 4: Alcohol PR	Inactive lever responses	Males	6.82 ± 1.62	3.64 ± 0.91	4.40 ± 0.91	**2.27 ± 0.59**	
		Females	5.50 ± 1.64	5.17 ± 1.66	8.25 ± 2.95	**1.92 ± 0.65**	
Exp 4: Alcohol + quinine PR	Inactive lever responses	Males	4.27 ± 1.36	3.27 ± 1.41	3.82 ± 1.06		
		Females	4.42 ± 0.90	4.33 ± 0.86	2.00 ± 0.28		
Exp 5: Seeking phase	Inactive lever responses	Males^a^	2.99 ± 0.78			2.36 ± 0.65	0.42 ± 0.26
		Females^a^	5.17 ± 0.61			3.75 ± 0.86	3.25 ± 1.00
Exp 5: Reinit. phase	Inactive lever responses	Males	1.09 ± 0.42			1.09 ± 0.09	0.08 ± 0.08
		Females	1.00 ± 0.35			1.25 ± 0.57	1.42 ± 1.07

*Note:* Bold indicates a significant main effect of dose, with post hoc analyses finding the dose was differences from controls. *p* < 0.05.

^a^ Indicates the main effect of sex.

In sucrose self‐administration, there were no effects of RTI‐122 on sucrose lever responding (Figure 3D) or sucrose intake (Table 1). The three‐way RM ANOVA of cumulative lever responses over time found no effects of RTI‐122 on lever responding, with only a main effect of time [F(5,15) = 37.80, *p* < 0.0001]. Locomotor rate was reduced [F(3,57) = 3.95, *p* < 0.05] at the 10 mg/kg dose (*p* < 0.05), and females had higher overall locomotion than males [F(1,19) = 35.01, *p* < 0.0001]. There were no differences in inactive lever responses (Table 1).

### 3.4 Experiment 4: Progressive Ratio

**FIGURE 4:**
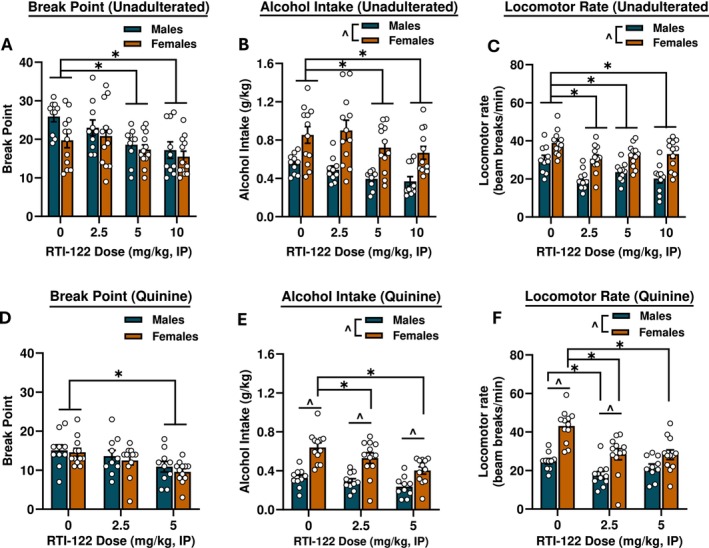
RTI‐122 reduced breakpoint in both unadulterated and quinine adulterated alcohol. The 5 and 10 mg/kg doses of RTI‐122 significantly reduced break point (A) and total intake (B) in progressive ratio self‐administration sessions while also reducing locomotor rate at all doses tested (C). When alcohol was adulterated with quinine, the 5 mg/kg dose reduced break point (D). A significant interaction found that intake was reduced specifically in females (E). Locomotor activity was again generally reduced by RTI‐122 (F). Males, *n* = 13; females, *n* = 12. *effect of treatment, ^effect of sex: *p* < 0.05.

To evaluate motivation to self‐administer alcohol, a progressive ratio schedule was initiated. RTI‐122 significantly reduced the break point in lever responses (Figure 4A) and overall alcohol intake (Figure 4B) for unadulterated alcohol [F(3,60) = 14.13, *p* < 0.0001; F(3,60) = 11.62, *p* < 0.0001; respectively], with reductions at the 5 and 10 mg/kg doses. For alcohol intake (g/kg), there was a main effect of sex with females consuming more alcohol by weight relative to the males [F(1,20) = 14.80, *p* < 0.001]. Females also had higher locomotor rates [F(1,20) = 28.10, *p* < 0.0001] and RTI‐122 reduced locomotion [F(3,60) = 12.00, *p* < 0.0001] at all doses tested (*p* < 0.01; Figure 4C). There was a main effect of dose on inactive lever responding [F(3,63) = 4.31, *p* < 0.01] which was reduced at the highest dose only (*p* < 0.05; Table 1).

When alcohol was adulterated with quinine to assess aversion‐resistant alcohol consumption, RTI‐122 reduced break point [F(2,40) = 13.72, *p* < 0.0001] at the highest dose tested, 5 mg/kg (*p* < 0.0001; Figure 4D). Analysis of adulterated alcohol intake found the main effects of dose [F(2,40) = 15.93, *p* < 0.0001], sex F(1,20) = 25.47, *p* < 0.0001 and a significant interaction [F(2,40) = 3.26, *p* < 0.0001] where intake was reduced in females at both the 2.5 and 5 mg/kg doses (*p* < 0.05) and males and females differed at all doses (*p* < 0.01). Locomotor rate was also significantly reduced by RTI‐122 as there was a main effect of dose [F(2,40) = 20.30, *p* < 0.0001], sex [F(1,20) = 21.82, *p* < 0.0001], as well as an interaction [F(2,40) = 5.075, *p* < 0.05]. Females had decreased locomotor behaviour relative to vehicle at both 2.5 and 5 mg/kg (*p* < 0.0001), while males only showed a decrease at the lower dose (*p* < 0.05), and the sexes differed after vehicle and 2.5 mg/kg injections (*p* < 0.01). In sum, RTI‐122 was effective at reducing motivation to self‐administer alcohol similarly whether the alcohol was adulterated with quinine or unadulterated. There were no differences in inactive lever responses (Table 1).

### 3.5 Experiment 5: Yohimbine‐Induced Reinstatement

**FIGURE 5:**
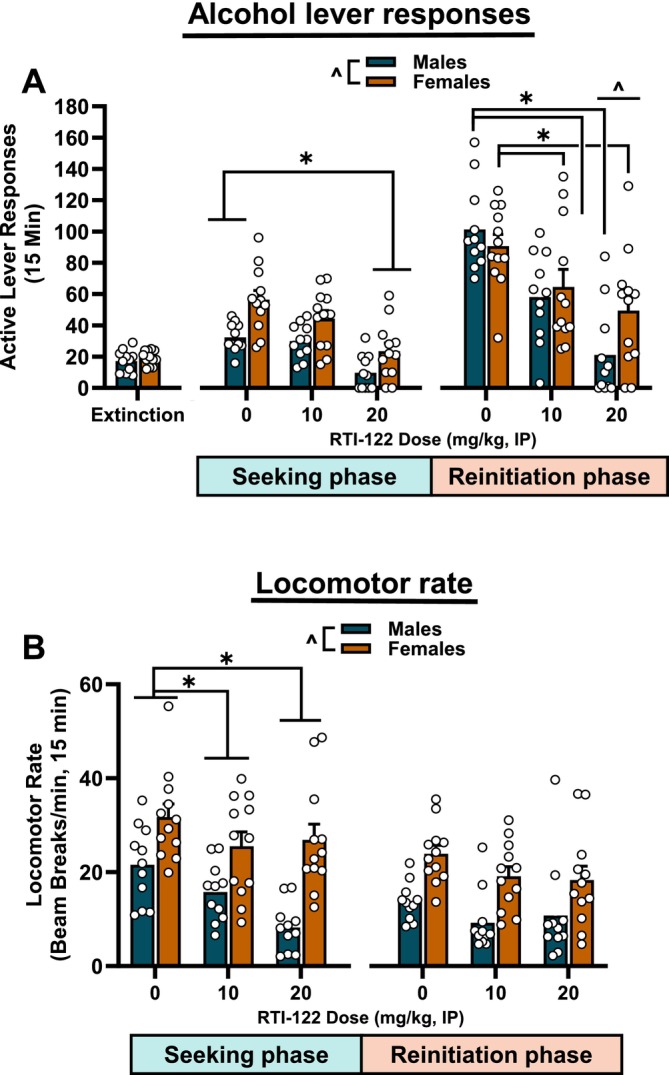
RTI‐122 reduced seeking and reinitiation of drinking in a two‐phase yohimbine‐induced reinstatement test. The 20 mg/kg dose of RTI‐122 reduced lever responses in the 15‐min seeking phase of the reinstatement test, akin to a traditional reinstatement test (A, middle panel). In the immediately subsequent 15‐min reinitiation of drinking phase where lever responding was reinforced, both the 10 and 20 mg/kg doses reduced lever responses (A, right panel). Locomotor rates were also reduced in the seeking phase (B, left panel) but not in the reinitiation phase (B, right panel). Males, *n* = 13; females, *n* = 12. *effect of treatment, #effect of dose in males, ! effect of dose in females, ^effect of sex: *p* < 0.05.

Experiment 5 examined whether RTI‐122 would reduce pharmacological stress‐induced reinstatement using a two‐phase reinstatement test. The first phase represented traditional reinstatement where lever responses are not reinforced, but the response‐contingent cues are presented (seeking phase). The second phase allowed for drinking, similar to a standard self‐administration session (reinitiation phase). Reinstatement behaviour was confirmed via two‐way ANOVA comparing the average of the prior three extinction sessions with the seeking‐phase vehicle group which found the main effects of reinstatement [F(1,21) = 72.85, *p* < 0.0001], sex [F(1,21) = 10.72, *p* < 0.01 and an interaction [F(1,21) = 12.91, *p* < 0.01] with both sexes demonstrating reinstatement (*p* < 0.01). Each phase was analysed separately. In the seeking phase (Figure 5A), RTI‐122 reduced seeking behaviour as supported by a two‐way RM ANOVA which found the main effects of sex [F(1,21) = 14.82, *p* < 0.001] and dose [F(2,42) = 25.56, *p* < 0.0001], with an overall reduction at 20 mg/kg (*p* < 0.0001). There was no sex by dose interaction. RTI‐122 also reduced self‐administration during the reinitiation of drinking phase (Figure 5A), as supported by a significant main effect of dose [F(2,42) = 42.27, *p* < 0.0001] and an interaction between dose and sex [F(2,42) = 4.37, *p* < 0.05]. Responding was reduced at all doses for both sexes (*p* < 0.05), and males responded less than females at the 20 mg/kg dose (*p* < 0.05). Inactive lever responding was low; during the seeking phase, there were main effects of sex [F(1,21) = 9.80, *p* < 0.01] and dose [F(2,42) = 4.97, *p* < 0.05] where females had more lever presses than males, and responding was lower than controls at the highest 20 mg/kg dose overall (*p* < 0.01; Table 1). During the seeking phase, for locomotion, there were main effects of dose [F(2,42) = 9.95, *p* < 0.001] and sex [F(1,21) = 20.44, *p* < 0.001], where females had greater locomotion than males and both RTI‐122 doses reduced activity (*p* < 0.05) (Figure 5B). During the reinitiation phase, only a significant sex effect was observed [F(1,21) = 27.31, *p* < 0.0001] where once again females had greater locomotion than males. Overall, RTI‐122 was effective at reducing stress‐induced (yohimbine) alcohol relapse‐like behaviour, as measured by alcohol‐seeking and the reinitiation of drinking.

## 4 Discussion

In the present experiments, we found RTI‐122 selectively reduced alcohol intake across different drinking assessments in mice and rats, aligning with previous findings from the characterization of our initial GPR88 compound [9]. In mice, RTI‐122 caused GPR88‐specific reductions in two‐bottle choice alcohol intake, and P‐rats showed reductions in alcohol self‐administration. Importantly, the GPR88‐specific mechanism was confirmed by the lack of effect in *Gpr88* KO mice, and RTI‐122 had no effects on water intake in mice or sucrose self‐administration in P‐rats, indicating that receptor specificity and its effects are specific to alcohol intake rather than general reward consumption. Progressive ratio experiments found that RTI‐122 reduced motivation to self‐administer alcohol at similar doses as self‐administration, regardless of whether the alcohol was adulterated with quinine or not, and yohimbine‐induced reinstatement was less sensitive and required a higher dose for efficacy. Overall, these findings suggest that RTI‐122 and GPR88 agonism, in general, could serve as effective and specific treatment targets for reducing alcohol‐related behaviours across several domains.

RTI‐122 reduced locomotor activity in C57BL/6 male mice at the two highest doses tested (10 and 20 mg/kg), which was particularly pronounced in the first 20 min of the 60‐min test session. This is not unexpected, as *Gpr88* KO results in hyperactivity [6, 17], and we previously found that RTI‐13951‐33 dose‐dependently reduced locomotor activity [9]. In those experiments overall, we noted a temporal dissociation between the locomotor effects and alterations in alcohol intake, with locomotor reductions being most pronounced early in the time course while reductions in drinking were maintained for up to 4 h. Thus, while the impact of RTI‐122 on locomotor activity at higher doses may limit its use at those levels, its ability to reduce alcohol intake independently of these effects highlights its potential as a targeted therapy for AUD.

Following the locomotor activity tests, a two‐bottle choice study was conducted to assess alcohol intake in both male and female WT or *Gpr88* KO mice, with bottle readings taken at 4 h and at the end of the session at 24 h. In male WT mice, RTI‐122 significantly reduced alcohol intake at both 10 and 20 mg/kg when assessed at the 4‐h mark, matching previous findings where it reduced intake in a 4‐h drinking‐in‐the‐dark (DID) design [11], and the effect was maintained at 24 h. Female WT mice required a higher dose (20 mg/kg) for a significant reduction in alcohol intake at 4 h. Critically, in both males and females, RTI‐122 did not reduce drinking in the *Gpr88* KO mice, confirming that effects are mediated by the GPR88 receptor. The female WT mice requiring a higher dose to reduce alcohol intake may be suggestive of a general reduced sensitivity to RTI‐122, which could be evaluated by testing locomotor activity in females as well.

In the male and female P‐rats, RTI‐122 reduced alcohol self‐administration at both 5 and 10 mg/kg. Self‐administration of sucrose was unaffected by RTI‐122, confirming that the reduction in alcohol reinforced responding reflects a specific effect on alcohol reinforcement rather than a general decrease in reward motivation. The 10 mg/kg dose decreased locomotor rate during the self‐administration session regardless of reinforcer but only reduced lever responding during alcohol self‐administration, suggesting that the observed reductions in alcohol intake are likely not solely due to locomotor suppression at this dose.

In the progressive ratio experiments, RTI‐122 reduced motivation to self‐administer alcohol, as both 5 and 10 mg/kg doses lowered breakpoints in males and females. This finding suggests that reduced motivation may be one mechanism by which GPR88 agonism attenuates alcohol intake. When alcohol was adulterated with quinine to assess drinking despite negative consequences, overall responding decreased, and RTI‐122 reduced the break point at 5 mg/kg but not 2.5 mg/kg. Overall, these findings indicate that RTI‐122 reduces alcohol consumption regardless of the alcohol solution's palatability or aversiveness, likely through changes in motivational processes. Within the context of alcohol use, this highlights RTI‐122’s potential as a broad behavioural modulator across varying conditions.

In the final experiment, the effects of RTI‐122 were assessed on yohimbine‐induced reinstatement following a period of extinction. Yohimbine acts as a pharmacological stressor via norepinephrine release in the brain, reliably inducing stress‐ and anxiety‐like responses and inducing reinstatement of drug seeking [18]. GPR88, known for its role in modulating anxiety [6, 12], may therefore influence stress‐induced reinstatement through its effects on neural circuits involved in both anxiety and reward processing. During the seeking phase, which mirrors traditional reinstatement (i.e., lever responses result in cue presentation but no reinforcer delivery), RTI‐122 reduced lever responding at the 20 mg/kg dose in both sexes but had no effect at the 10 mg/kg dose. Interestingly, the 10 mg/kg dose reduced alcohol self‐administration (Figure 3A) and break point in the progressive ratio task (Figure 4A). This discrepancy was unexpected, as both tasks assess motivation, albeit in different ways. In the progressive ratio task, there is continuous access to the reinforcer with increasing effort required, whereas seeking during reinstatement testing occurs in the absence of alcohol and after a period of abstinence. It is possible that the abstinence period involves different circuitry or induces a more resilient motivational drive, or it may be that GPR88 is not critically involved in stress‐induced reinstatement. Further research is needed to clarify the role of GPR88 in these distinct motivational processes and how each may need targeting with different dose ranges. The second phase of the reinstatement test is akin to self‐administration, and in this phase, both 10 and 20 mg/kg doses reduced self‐administration, consistent with the initial self‐administration assessment (Figure 3A). Together, these findings suggest that RTI‐122 is ultimately effective at reducing stress‐induced reinstatement and reinitiation of drinking, which may have relevance for the treatment of relapse‐like behaviour.

Overall, some sex differences were found in these studies. In the two‐bottle choice experiments, the female WT mice requiring a higher dose to reduce alcohol intake may be suggestive of a general reduced sensitivity to RTI‐122. In self‐administration, no sex‐dependent effects of RTI‐122 were found; however, the lowest 2.5 mg/kg dose appeared to have some efficacy in females, though no interaction was found and post hoc analyses could not confirm this. It would be unexpected if female rats were more sensitive to GPR88 agonism while female mice (Experiment 2) appeared to be less sensitive, though this should be interpreted with caution given the lack of effect and that these drinking models have major differences in both duration (30 min vs 4 h) and overall design. Additionally, we previously found that the GPR88 agonist RTI‐13951‐33 reduced self‐administration in female Long Evans rats at 10 and 20 mg/kg but not at 5 mg/kg [10], while RTI‐122 was effective at 5 mg/kg, indicating greater efficacy. This was also seen in binge‐like alcohol consumption in mice, where RTI‐122 at 10 mg/kg matched the effects of RTI‐13951‐33 at 30 mg/kg [10]. However, a more comprehensive comparison across sex and strain is needed to determine the consistency of these effects. Interestingly, no sex differences in RTI‐122 efficacy emerged in either the progressive ratio or reinstatement experiments. This may suggest that sensitivity differences between males and females involve factors other than motivation.

In both of the progressive ratio assessments and in reinstatement, RTI‐122 induced a reduction in locomotor behaviour during the sessions at all doses tested even though in both experiments effects on motivation were specific to higher doses. Interpretation of locomotor activity across operant tasks is challenging, particularly in sessions that deviate from trained reinforcement contingencies, where reduced movement may reflect disengagement or responses to unmet expectations rather than behavioural suppression. However, as reduced alcohol lever responding without a reduction in locomotion at the 5 mg/kg dose was observed in Experiment 3, these results further suggest that the effects of RTI‐122 on alcohol‐operant related behaviours are not solely due to reductions in general activity. This interpretation is further supported by the FR2 self‐administration data, where reductions in locomotion occurred only at the highest dose (10 mg/kg), matching the pattern observed in Experiment 1, which used free exploration in a dedicated locomotor chamber to more directly assess general activity.

Overall, our findings suggest that GPR88 agonism with RTI‐122 primarily affects the motivational aspects of alcohol consumption. It is efficacious in reducing the amount of home cage drinking and operant alcohol self‐administration, and our reinstatement findings suggest that higher doses may be capable of reducing the initial desire to consume alcohol. Indeed, we previously showed that RTI‐13951‐33 diminished the rewarding effects of alcohol in a place preference test, which with the present data could suggest a similar mechanism as the FDA‐approved treatment naltrexone [9]. In contrast, acamprosate is specifically effective at maintaining abstinence [19], and RTI‐122 may be capable of addressing both therapeutic goals. The cell‐type specificity of GPR88 expression, which is primarily expressed in both D1‐type and D2‐type GABAergic medium spiny neurons in the striatum, likely plays a key role in the effects of RTI‐122 and logically aligns with our findings that suggest a general reduction in motivation. Conditional knockouts found that D1 and D2 neurons modulate different sets of behaviours [5, 20], which may suggest opportunity for greater specificity in targeting AUD. While these findings are consistent with a striatal mechanism of action, GPR88 is also expressed in extrastriatal brain regions implicated in alcohol intake and self‐administration, including the amygdala, ventral pallidum, insular cortex, piriform cortex and somatosensory cortex [21, 22]. Given the established roles of these regions in cue‐driven behaviour, reward valuation, interoception and sensory integration, future region‐ or circuit‐specific studies may clarify how coordinated interactions between striatal and extrastriatal circuits contribute to the therapeutic effects of GPR88 agonism on alcohol‐related behaviours. Together, these findings support the continued evaluation of RTI‐122 as a promising research compound for advancing the development of more effective and precise treatments for AUD.

## Author Contributions

D.F.L.: conducted rat experiments, analyzed data, prepared figures, prepared initial manuscript draft and contributed to manuscript drafting and editing. W.L.: conducted rat experiments, analyzed data, prepared initial figures, and contributed to manuscript drafting and editing. S.B.H.: conducted mouse experiments, analyzed data, prepared figures, and contributed to manuscript drafting and editing. V.L.C.: conducted rat experiments, analyzed data. K.J.V.V.: conducted rat experiments, analyzed data. M.M.: conducted mouse experiments, analyzed data. I.G.O.: conducted mouse experiments, analyzed data. E.D.: conducted mouse experiments, analyzed data, prepared figures, and contributed to manuscript drafting and editing. M.T.R.: synthesized RTI‐122 for behavioral experiments. M.N.: conducted mouse experiments, analyzed data. B.L.K.: contributed to the design of mouse experiments. C.J.: project conceptualization and funding acquisition, managed project across collaborators, contributed to manuscript writing and editing. J.B.: project conceptualization and funding acquisition, project supervision, contributed to manuscript writing and editing.

## Conflicts of Interest

The authors declare no conflicts of interest.

## Supporting information


**Figure S1:** RTI‐122 does not affect water consumption in two‐bottle choice test. *Gpr88* knockout (*Gpr88* KO) and control (*Gpr88* WT) mice underwent the IA20%‐2bc procedure for 6 weeks. On test days, male (A–D) and female (E–H) mice received either vehicle or RTI‐122 at doses of 10 mg/kg (males: A–B; females: E–F) or 20 mg/kg (males: C–D; females: G–H), administered 60 min prior to the start of the 24‐h session. Water intake was measured at 4 and 24 h. Data are presented as mean ± S.E.M.
**Figure S2.** Course of extinction and test days for Experiment 5. To confirm extinction, a two‐way ANOVA comparing the first 16 days of extinction found a main effect of day [F(15, 240) = 45.42, *p* < 0.0001]. Post hoc analysis showed that extinction day 1 had greater lever responding than all subsequent days. There were also no significant differences between any days from extinction days 5 through 16, indicating that responding remained consistently low and stable throughout this period. Posttesting training days are shown but were not included in the analysis. *Significantly different from extinction day 1, *p* < 0.05.

## Data Availability

Data are available on request from the authors.
