# Supplementary material for: The GPR88 Agonist RTI‐122 Reduces Alcohol‐Related Motivation and Consumption
Source: Addict Biol. 2025 Jun 19;30(6):e70058. doi: 10.1111/adb.70058 (PMC12178211; doi:10.1111/adb.70058)
Supplement: Supplementary file 1 — Figure S1: RTI‐122 does not affect water consumption in two‐bottle choice test. Gpr88 knockout (Gpr88 KO) and control (Gpr88 WT) mice underwent the IA20%‐2bc procedure for 6 weeks. On test days, male (A–D) and female (E–H) mice received either vehicle or RTI‐122 at doses of 10 mg/kg (males: A–B; females: E–F) or 20 mg/kg (males: C–D; females: G–H), administered 60 min prior to the start of the 24‐h session. Water intake was measured at 4 and 24 h. Data are presented as mean ± S.E.M. Figure S2. Course of extinction and test days for Experiment 5. To confirm extinction, a two‐way ANOVA comparing the first 16 days of extinction found a main effect of day [F(15, 240) = 45.42, p < 0.0001]. Post hoc analysis showed that extinction day 1 had greater lever responding than all subsequent days. There were also no significant differences between any days from extinction days 5 through 16, indicating that responding remained consistently low and stable throughout this period. Posttesting training days are shown but were not included in the analysis. *Significantly different from extinction day 1, p < 0.05. [file ADB-30-e70058-s001.pdf]

Supplemental Figure 1

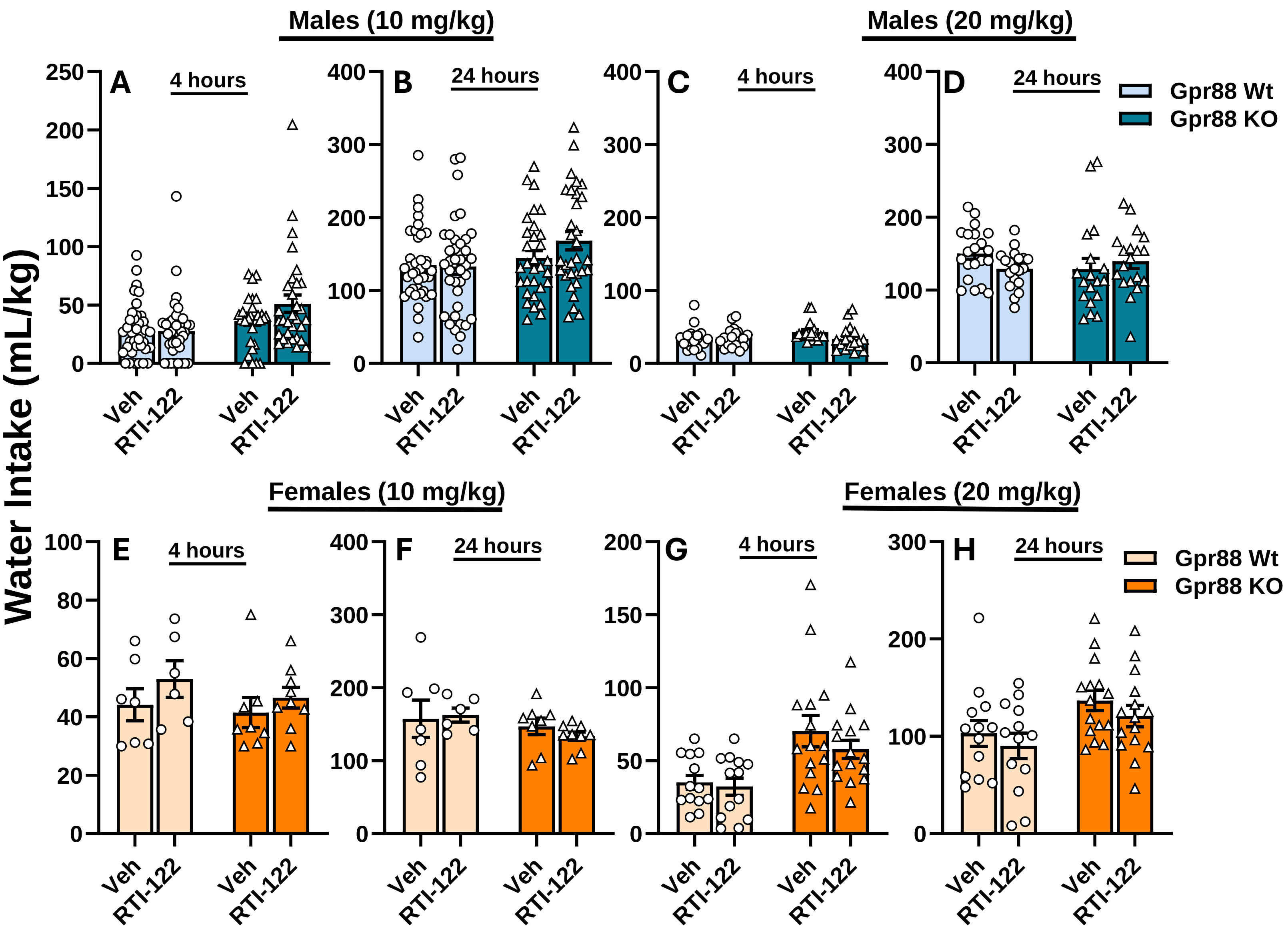

Supplemental Figure 1: RTI-122 does not affect water consumption in two bottle choice test. *Gpr88* knockout (*Gpr88* KO) and control (*Gpr88* WT) mice underwent the IA20%-2BC procedure for 6 weeks. On test days, male (A–D) and female (E–H) mice received either vehicle or RTI-122 at doses of 10 mg/kg (males: A–B; females: E–F) or 20 mg/kg (males: C–D; females: G–H), administered 60 minutes prior to the start of the 24-h session. Water intake was measured at 4 and 24 hours. Data are presented as mean ± S.E.M.

Supplemental Figure 2

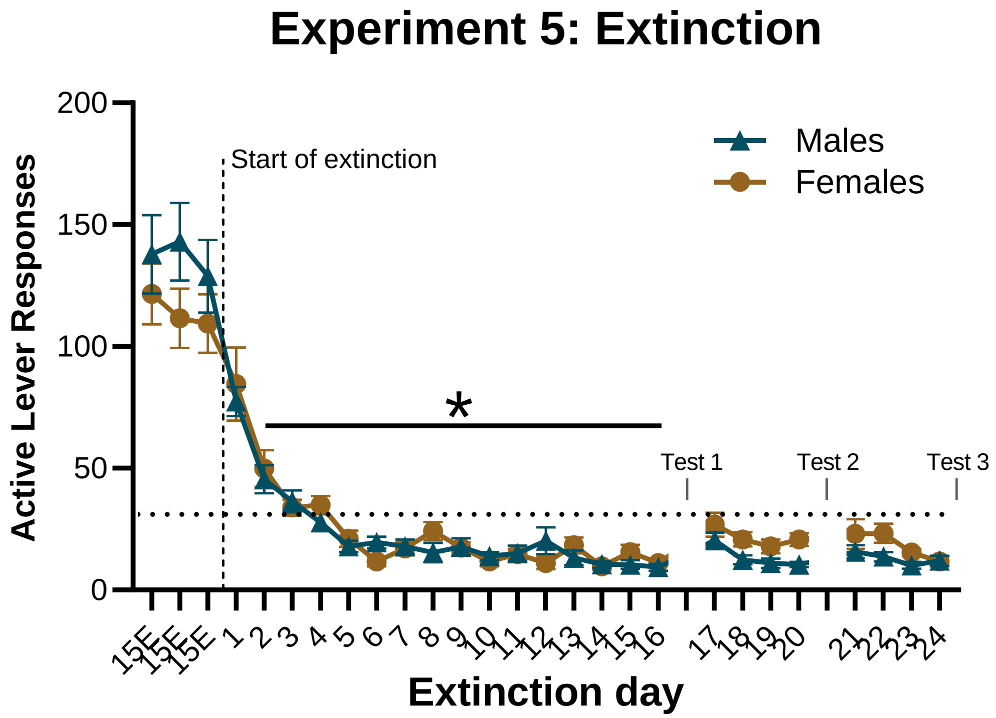

Supplemental Figure 2. Course of extinction and test days for Experiment 5. To confirm extinction, a two-way ANOVA comparing the first 16 days of extinction found a main effect of day [$F(15, 240) = 45.42$, $p<0.0001$]. Post-hoc analysis showed that extinction day 1 had greater lever responding than all subsequent days. There were also no significant differences between any days from extinction days 5 through 16, indicating that responding remained consistently low and stable throughout this period. Post-testing training days are shown but were not included in the analysis. *Significantly different from extinction day 1, $p<0.05$.
